# Data for behavioral results and brain regions showing a time effect during pair-association retrieval

**DOI:** 10.1016/j.dib.2016.06.054

**Published:** 2016-07-06

**Authors:** Koji Jimura, Satoshi Hirose, Hiroyuki Wada, Yasunori Yoshizawa, Yoshio Imai, Masaaki Akahane, Toru Machida, Ichiro Shirouzu, Yasuharu Koike, Seiki Konishi

**Affiliations:** a Department of Physiology, The University of Tokyo School of Medicine, 7-3-1 Hongo, Bunkyo-ku, Tokyo, Japan; b Department of Biosciences and Informatics, Keio University, 3-14-1 Hiyoshi, Kohoku-ku, Yokohama, Japan; c Precision and Intelligence Laboratory, Tokyo Institute of Technology, 4259 Nagatsuta-cho, Midori-ku, Yokohama, Japan; d Department of Physiology, Juntendo University School of Medicine, 2-1-1 Hongo, Bunkyo-ku, Tokyo, Japan; e Department of Radiology, NTT Medical Center Tokyo, 5-9-22 Higashi Gotanda, Shinagawa-ku, Tokyo, Japan

## Abstract

The current data article provides behavioral and neuroimaging data for the research article "Relatedness-dependent rapid development of brain activity in anterior temporal cortex during pair-association retrieval” (Jimura et al., 2016) [1]. Behavioral performance is provided in a table. Fig. 2 of the article is based on this table. Brain regions showing time effect are provided in a table. A statistical activation map for the time effect is shown in Fig. 3C of the article.

## Specifications Table

**Table:** 

Subject area	*Neuroscience, psychology*
More specific subject area	*Human long-term memory*
Type of data	*Tables*
How data was acquired	*Functional MRI, task behavior*
Data format	*Analyzed*
Experimental factors	*Brain imaging and behavior*
Experimental features	*Pair-association task*
Data source location	*Tokyo, Japan*
Data accessibility	*Data is within this article*

## Value of the data

• Behavioral results can be used to see performance of the pair association task.
• List of coordinates in standard space are used to see brain regions showing the time effect.
• The data can be used for any meta-analytic purpose.

## 1 Data

Behavioral results for the performance of the pair association task are shown in Table 1. Brain regions showing significant time effect are listed in Table 2.

## 2 Experimental design, materials and methods

Group-level mean and standard errors of the mean (SEM) were calculated for behavioral performance of the pair-association task [1]. The performance was calculated for each experimental condition. Brain regions were identified showing voxel-level significant effect of time in a group analysis (*P*<.05, corrected for multiple comparisons across the whole brain with family-wise error rate). Coordinates are listed in the order of statistical significance level. Full descriptions are available in the article.

## Figures and Tables

**Table 1:** Behavioral results [mean (SEM)].

Time	Category	Reaction times (ms)	Percentage remember trial
Remote	Related	941 (33.4)	86.3 (15.7)
	Unrelated	957 (29.8)	81.1 (22.2)
	Novel	1026 (31.3)	56.1 (24.8)
Recent	Related	773 (24.1)	93.7 (12.4)
	Unrelated	816 (31.6)	89.6 (19.1)
	Novel	850 (28.1)	90.0 (13.2)

**Table 2:** Brain regions showing a time effect in a two-way ANOVA.

Region		*F-*value	*x*	*y*	*z*	Contrast
Frontal	Anterior insula	126.8	34	26	−2	RM>RC
	Lateral inferior	99.2	50	28	22	RM>RC
	Dorsolateral	79.6	−42	50	−2	RM>RC
	Dorsolateral	75.3	−50	20	30	RM>RC
	Dorsolateral	71.2	2	22	62	RM>RC
	Lateral inferior	69.2	−48	34	−2	RM>RC
	Dorsolateral	67.7	44	10	32	RM>RC
	Anterior insula	65.4	−30	28	−4	RM>RC
	Lateral orbital	61.0	−42	32	−18	RM>RC
	Dorsolateral	58.7	−2	26	48	RM>RC
	Dorsolateral	57.8	−8	16	50	RM>RC
	Dorsolateral	55.8	−2	48	40	RM>RC
	Lateral orbital	47.2	−38	46	−10	RM>RC
	Lateral inferior	44.4	−40	6	22	RM>RC
	Lateral inferior	43.4	42	22	28	RM>RC
Others	Caudate	77.4	−12	10	12	RM>RC
	Thalamus	66.1	−6	−10	2	RM>RC
	Cerebellum	52.3	0	−54	−42	RM>RC
	Cerebellum	44.9	8	−56	−46	RM>RC
