# Supplementary material for: Data for behavioral results and brain regions showing a time effect during pair-association retrieval
Source: Data Brief. 2016 Jul 6;8:891–3. doi: 10.1016/j.dib.2016.06.054 (PMC4961795; doi:10.1016/j.dib.2016.06.054)
Supplement: Supplementary file 1 — Supplementary material [file mmc1.docx]

The authors declare no conflicts of interests.
